# Effects of Moderate- versus Mixed-Intensity Training on VO_2_peak in Young Well-Trained Rowers

**DOI:** 10.3390/sports9070092

**Published:** 2021-06-25

**Authors:** Timo Kirchenberger, Sascha Ketelhut, Reinhard G. Ketelhut

**Affiliations:** 1 Campus Virchow-Klinikum, Charité-Universitätsmedizin Berlin, 13353 Berlin, Germany; timo.kirchenberger@charite.de; 2 Institute of Sport Science, University of Bern, 3012 Bern, Switzerland; 3 Medical Center Berlin, 10559 Berlin, Germany; r.ketelhut@t-online.de

**Keywords:** rowing performance, endurance training, young athletes, maximal oxygen consumption, high-intensity interval training

## Abstract

The effects of moderate-intensity continuous training (MICT) and a combination of MICT and high-intensity interval training (HIIT) on rowing performance and VO_2_peak were investigated in young athletes. Seventeen well-trained rowers (aged 15 ± 1.3 years) were randomly allocated to an intervention (IG) (*n* = 10) and control group (CG) (*n* = 7). During 8 weeks, both groups took part in the regular rowing training (3×/week MICT, 70–90 min, 65–70% of HRpeak + 2×/week resistance training). The IG completed an additional high-intensity interval training twice weekly (2 × 4 × 2 min at ≈95% of HRpeak, 60 s rest). Instead of the HIIT, the CG completed two more MICT sessions (70–90 min, 65–70% of HRpeak). Before and after the intervention, a 2000 m time trial and an exercise test were performed. The IG showed a significant improvement (*p* = 0.001) regarding the absolute rowing time in the graded exercise test. Furthermore, the intervention group showed a significant increase in relative VO_2_peak (*p* = 0.023), a significant increase in absolute VO_2_peak (*p* = 0.036), and a significant improvement in the 2000 m time trail (*p* = 0.003). No significant changes could be detected in the CG. The interaction effects were not significant. A mixed-intensity training, including HIIT, was beneficial on rowing performance and VO_2_peak in highly trained athletes.

## 1. Introduction

The literature confirms that high-intensity interval training (HIIT) improves performance and aerobic capacity in high-risk patients [1,2], as well as in healthy and endurance-trained athletes [3,4,5]. HIIT produced similar or even superior changes in cardiorespiratory fitness in different populations compared to moderate-intensity continuous training (MICT) despite significantly reduced time commitment [1]. Thus, HIIT seems to be a promising and time-efficient alternative or addition to commonly used MICT, especially in high-volume sports.

Although numerous studies have highlighted the effectiveness of HIIT, and different HIIT protocols are widely used in different sports, some sports are still neglecting this trend.

Rowing training protocols traditionally display a marked emphasis on high training volumes with intensities below the lactate threshold [6,7] despite competing at much higher intensities [8]. Still, little overall focus is placed on higher training intensities, which are considered necessary to develop maximal aerobic power [6]. This may be due in part to the fact that studies evaluating the effects of HIIT in well-trained rowers are sparse. So far, only a few studies have examined the effectiveness of HIIT on endurance performance in this cohort [6,7,9,10].

To the best of our knowledge, controlled studies that included HIIT in the training protocol of junior rowers have not been conducted. However, time-efficient training is particularly convincing for young athletes who must cope with the double burden of school and competitive sports. In addition, children and adolescents seem to have optimal preconditions for HIIT, as they show a higher fatigue resistance and a fast recovery after exercises with high to maximum intensities compared to adults [11,12].

A retrospective study showed that rowers who are internationally successful as seniors tend to do a higher amount of their total endurance exercises at low intensity and very high intensity as juniors compared to their peers [13]. Accordingly, the combination of large amounts of long-distance training and HIIT can represent a best practice model for developing endurance performance, even in young rowers.

Therefore, the present study examined the effects of MICT and a combination of MICT and HIIT on rowing performance and VO_2_peak in young athletes.

## 2. Materials and Methods

### 2.1. Subjects

Seventeen well-trained male rowers (age 15 ± 1.3 years; BMI 22.1 ± 2.6 kg/m^2^; VO_2_peak 59.0 ± 3.9 mL/kg/min (Table 1)) who competed at the national and international level were included in the study. All had a training history of 5 to 8 years, were non-smokers, and were free of known injuries and acute or chronic illnesses as assessed by the medical history questionnaire. All athletes and their legal guardians gave written informed consent to participate in the study. The experimental procedures of the study were approved by the institutional research committee (Charité-Universitätsmedizin Berlin, Berlin, Germany, EA2/093/18) and carried out according to international standards [14].

### 2.2. Study Design

The study was conducted as a parallel-arm randomized controlled trial. The examinations of the participants took place at baseline (pre) and after (post) an eight-week training period. Following the baseline assessment, the athletes were randomly assigned to an intervention (IG) (*n* = 10) and a control group (CG) (*n* = 7). The randomization was carried out by using a computer-generated random number table.

For each participant, the testing procedures (pre and post) were carried out in the same order and at the same time of day on the same day of the week. Specially trained staff performed the tests under standardized conditions. The same examiner obtained the pre- and post-parameters with the same devices. All tests were performed in the same temperature-controlled laboratory (22.5 ± 1 °C).

The athletes reported to the laboratory of the Medical Center Berlin in a rested and fasting state. They were instructed to refrain from caffeinated and alcoholic beverages for at least 4 h prior to measurements.

On the first day, all completed a validated physical activity questionnaire, MoMo-AFB [15,16], to assess habitual physical activity apart from rowing training. Anthropometric measures were taken, and subjects completed a graded exercise test (GXT). On the second day (>48 h later) a 2000 m time trial (TT) was conducted. Both tests were performed on an air-braked rowing ergometer (Concept 2, Model D, Morrisville, VT, USA).

### 2.3. Incremental Exercise Test

On the first day, the participants completed a GXT until voluntary exhaustion. The test protocol was performed according to the guidelines of the German Rowing Association [17]. After a 5-min warm-up, the GXT began with 150 watts for lightweights (<65 kg) and 200 watts for heavyweights (>65 kg) and a stroke frequency of 18–20 strokes/min. Each stage lasted 4 min and was interspersed by 30 s of rest. After each stage, the workload was increased by 50 watts and 2 strokes/min. Following the fourth stage, the stroke frequency was freely selectable by the athletes. The drag factor was set to 130, as recommended by the Deutscher Ruder-Verband e.V. [17].

Heart rate (HR) was monitored by Polar HR-monitor (Polar Electro OY, Kempele, Finland), and HRpeak was the highest recorded value.

Expired air was continuously analyzed (breath-by-breath) for O_2_ and CO_2_ concentrations, using the Metalyzer 3B-R2 (Cortex Biophysik GmbH, Leipzig, Germany). The highest 30 s VO_2_ value was recorded as VO_2_peak. Before each test, gas analyzers were calibrated as per the manufacturer’s guidelines with gases of known composition. To calibrate respiratory volume, a 3 L–volume calibration syringe was used.

### 2.4. Two-Thousand-Meter Time Trial

The TT was performed on the same rowing ergometer as the GXT. The test-retest reliability of 2000 m TT on the Concept II rowing ergometer has previously been examined with well-trained rowers revealing a coefficient of variation of 2.0% [18]. The TT is an integral part of rowing performance monitoring; thus, all subjects were fully familiar with the test. Before the test, the subjects performed a 10-min self-selected warm-up. The time to complete the TT was recorded. The stroke frequency was freely selectable by each subject, and the drag factor was set to 130. To control psychological motivation, no verbal encouragement was given.

### 2.5. Intervention

The study was conducted during the preparation phase of the yearly training program. The IG and CG participated in the regular rowing training, which consisted of MICT (on a rowing ergometer) for 70–90 min at 65–70% HRpeak (3×/week) and strength training (2×/week). Both groups performed the same strength-training program throughout the intervention period. The training consisted of different exercises for the upper and lower body. Depending on the exercise, 3 or 4 sets of 2–10 repetitions at 70–92% of one-repetition max were performed.

In addition to this regular training, the athletes performed twice a week either an additional HIIT or MICT on a rowing ergometer.

**Figure 1:**
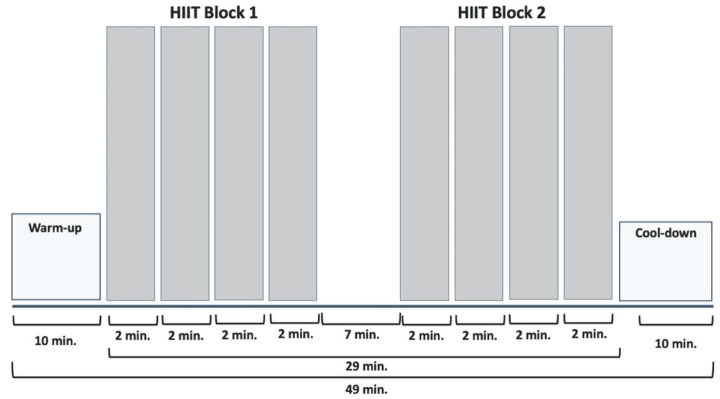
HIIT protocol of the intervention group.

The HIIT started with a 10-min moderate warm-up. Subsequently, 2 blocks of 4 × 2 min were applied at ≈95% of HRpeak interspersed with 1 min of passive rest. Between the 2 blocks, there was a 7-min passive break. The protocol culminated in 10 min of cooling-down (Figure 1). The total duration of the training was 49 min.

The MICT consisted of 70–90 min ergometer training at an intensity of 65–70% of the HRpeak. All ergometer training sessions were performed on the same device as the GXT. All training sessions were overseen and documented by the two coaches.

### 2.6. Statistical Analysis

Statistical analysis was performed by using IBM SPSS Statistics v. 25.0 (SPSS, Chicago, IL, USA). Paired samples *t*-tests were used to compare pre- and post-intervention values within groups. The Levene test was used to check the homogeneity of variance. Analysis of variance was performed to identify differences in the means, and a multiple pair-wise comparison was performed, using Bonferoni correction as a post hoc test. Level of significance was *p* < 0.05.

## 3. Results

**Figure 2:**
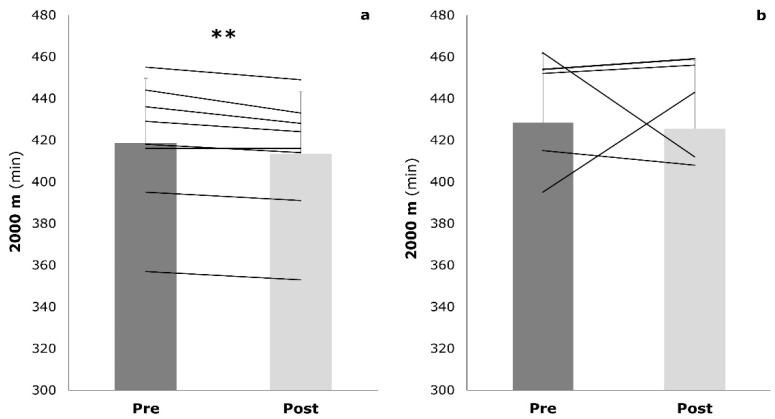
Two-thousand-meter time trial performance at baseline (pre) and post-training (post) in both the intervention group (**a**) and the control group (**b**). Values are mean ± SD; ** *p* < 0.01.

**Figure 3:**
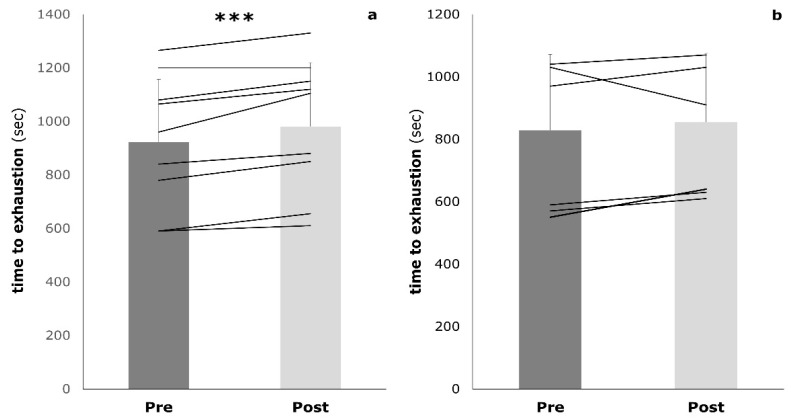
Time to exhaustion during the graded exercise test at baseline (pre) and post-training (post) in both the intervention group (**a**) and the control group (**b**); *** *p* < 0.001.

The subjects’ characteristics are presented in Table 1. The IG and the CG did not show any significant differences at baseline. During the intervention period, participants reported no significant changes in physical activity (pre: 16.3 ± 2.5 h/week vs. post: 16.6 ± 3.4 h/week). After the intervention, the IG showed a significant improvement in 2000 m TT (−5.3 ± 3.2 s, *p* = 0.003), whereas the CG showed no significant changes (−3.0 ± 3.5 s, *p* = 0.091) (Figure 2). The interaction effects between IG and CG were not significant (*p* = 0.239). The athletes of both groups improved their time to exhaustion in the GXT. However, only the mixed training was associated with a significant improvement (IG: +58.0 ± 38.2 s, *p* = 0.001; CG: +25.7 ± 67.3 s, *p* = 0.351) (Figure 3). Again, the interaction effects were not significant (*p* = 0.226).

**Figure 4:**
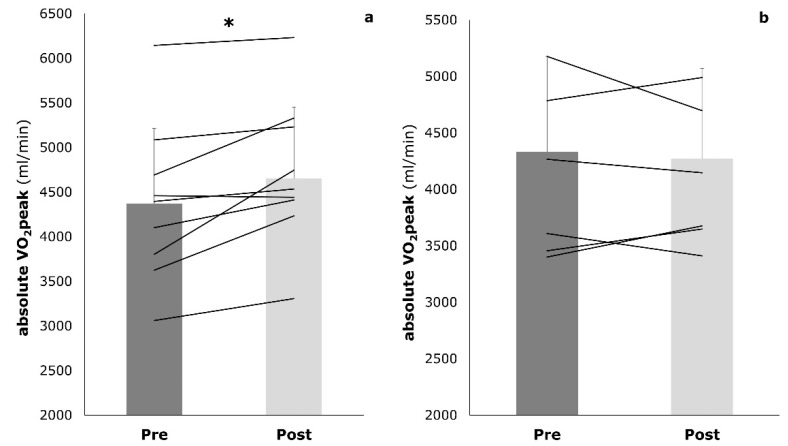
Absolute VO_2_peak (mL/min) at baseline (pre) and post-training (post) in both the intervention group (**a**) and the control group (**b**); * *p* < 0.05.

The IG showed a significant increase in absolute VO_2_peak compared to the initial examination (4371.30 ± 845.5 mL/min to 4651.7 ± 799.7 mL/min, *p* = 0.036). In the CG, there was a slight but not significant decrease in absolute VO_2_peak after the intervention (4331.6 ± 890.2 mL/min to 4271.3 ± 743.9 mL/min, *p* = 0.601) (Figure 4). The interaction effects approached statistical significance (*p* = 0.056).

**Figure 5:**
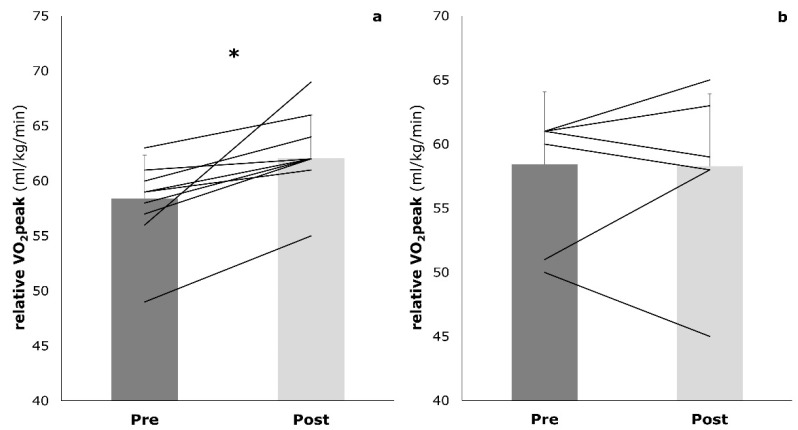
Relative VO_2_peak (mL/kg/min) at baseline (pre) and post-training (post) in both the intervention group (**a**) and the control group (**b**); * *p* < 0.05.

The mixed training was associated with a significant increase in relative VO_2_peak (58.4 ± 3.9 mL/kg/min to 62.1 ± 3.9 mL/kg/min, *p* = 0.023). No significant changes were detected for the CG (58.4 ± 5.7 mL/kg/min to 58.3 ± 6.4 mL/kg/min, *p* = 0.937) (Figure 5). The interaction effects did not reach statistical significance (*p* = 0.097).

## 4. Discussion

The data showed that well-trained young elite rowers achieved an advantage in the development of endurance performance by implementing HIIT in the regular training program.

The 2000 m TT performance, the relative and absolute VO_2_peak, and the performance in the GXT were significantly improved in the already well-trained athletes. In contrast, there was no significant change in the CG. The interaction effects for relative and absolute VO_2_peak were marginally significant.

Thus, HIIT presents an effective and time-efficient complement to conventional MICT in rowers.

This is in accordance with other studies in well-trained athletes with similar or superior effects of HIIT on endurance performance and VO_2_peak [3,5,19]. The research of HIIT on performance in elite rowers is limited.

In a 4-week crossover design, in well-trained rowers, HIIT induced greater improvements in 2000 m TT and relative VO_2_peak than the traditional rowing training in the control group [9]. Contrary to our study, where the IG conducted a mixed training, the HIIT group in the study by Diller et al. [9] exclusively participated in a HIIT program throughout the intervention period. This could possibly explain the stronger interaction effects compared to our study.

Like the present study, others have assessed the effects of replacing a part of the endurance-based program with HIIT sessions. Similar to our results, Ni Chèilleachair [6] showed greater improvements in the 2000 m TT performance and VO_2_peak in the mixed-intensity training group compared to the MICT group.

Ingham et al. [10], on the other hand, found no differences between a mixed-intensity protocol and a low-intensity protocol on 2000 m TT and VO_2_peak.

This is in line with the results from Stevens et al. [7], who compared a combined sprint interval training and endurance training protocol with an endurance-only protocol in trained rowers (aged 18–21 years). The authors found improvements in the 2000 m TT and peak power output in the HIIT group. However, neither group experienced any change in VO_2_peak, which the authors attribute to the fact that the regular training of the sample involved a large percentage of high-intensity exercise. Thus, the athletes could already have been accustomed to this kind of stimulus. The athletes in our study mainly performed MICT in their regular training. Therefore, the HIIT may have provided a more novel exercise stimulus for these athletes, consequently eliciting further performance improvements.

Even though the literature on HIIT in well-trained rowers confirms our results, it should be noted that, in previous studies, only adults were recruited. Unfortunately, the effects of HIIT in children and adolescents have been less studied than in adults. Most studies of HIIT in children and adolescents focus on cardio–respiratory fitness and health-related parameters in untrained or obese people [20,21]. The literature on young, trained athletes is rare. However, some studies in young athletes show an increase in VO_2_peak [22,23] in connection with HIIT. In young competitive swimmers, a 5-week HIIT resulted in an increase in VO_2_peak comparable to continuous training, with the training time for HIIT being only half as long as for MICT [24]. A recent review has analyzed various adaptations of HIIT exclusively in young athletes [25]. HIIT did not show any clear superiority in VO_2_peak compared to alternative training protocols. HIIT showed small mean effect sizes but a considerably higher percentage increase of VO_2_peak compared to alternative training programs, as well as small and large mean effect sizes on relevant aerobic and anaerobic performance parameters. Unfortunately, no studies have been carried out on young and well-trained rowers.

The mechanisms that are discussed regarding the effectiveness of HIIT are diverse. Regardless of the shorter training duration, various studies have shown similar reactions of the skeletal muscles after HIIT and MICT [26].

It is assumed that the training adjustments through HIIT and MICT trigger different intracellular stimuli (activation of AMP protein kinase, CaM kinase II, and p38-MAPkinase) but ultimately lead to the same metabolic cascades [27]. However, the improvements in performance seem to be mainly explained by the central oxygen supply and adaptations of the cardiovascular system and less by adaptations of the peripheral mechanisms [28]. The stronger effect of HIIT on the maximum oxygen uptake results from a larger left ventricular diastolic filling and thus a larger stroke volume due to a higher work rate and a longer exposure time close to VO_2_max [29,30]. This seems to be the case, in particular, in children and adolescents, in whom changes in VO_2_max are mainly due to an increased stroke volume due to an increased preload, a decreased afterload, and an enlarged heart [31].

This study complements the sparse literature on the effects of HIIT in young athletes. HIIT seems to be a time-efficient and appropriate training tool for enhancing aerobic performance. The inclusion of HIIT in the training program for young rowers appears very promising, as the demands of rowing at a high level require a considerable amount of time. In this regard, HIIT could be a suitable training method to improve endurance- parameters and leave enough time to improve sport-specific skills. By including HIIT in their training routine, athletes have the opportunity to train at the intensity levels they are competing at. Not only does this improve physical performance, but it can also affect technical skills, especially at the racing pace. This assumption is support by a recent study from Papandreou et al. [19]. They compared different physiological and performance variables after 8 weeks of MICT or HIIT in flat water kayak athletes. The authors could show that HIIT was more effective than MICT in improved paddling economy speed [19].

Especially for traditionally trained rowers who have mainly adapted to MICT, HIIT can offer a new type of exercise stimulus that could lead to further improvements in performance. Since HIIT is infinitely variable (intensity, duration, number of intervals, duration, and activity pattern during recovery), it can help modify the training stimuli throughout the season and promote ongoing adaptation.

Anecdotally, the participants in the HIIT group reported that the HIIT was more enjoyable than the MICT. This is supported by survey results from Kilpatrick [32] and highlights the potential of HIIT to increase motivation.

Our study gained meaningful insight into the benefits of HIIT for coaches and for future intervention studies in rowers. However, further research is warranted to optimize the HIIT protocol for well-trained young rowers, with regards to the manipulation of training variables (frequency, intensity, duration, recovery, etc.) and the organization with other training contents.

### Limitations

When interpreting these results, certain limitations must be considered. First, the sample size was relatively small. A larger sample size with a more homogeneous group can be beneficial. Second, accelerometry was not used to monitor everyday activities. However, physical activity questionnaires were used, and training protocols analyzed that showed no changes in either IG or CG. Moreover, well-trained, high-volume athletes are not expected to change physical activity during a training cycle. Third, we did not access recovery stress states during the intervention. It could be argued that HIIT could lead to early signs of fatigue and overtraining and thus reduce the effects. Analysis of recovery-stress states during HIIT interventions, using psychological and physiological methods, would be advisable in assessing the effects of HIIT.

In addition, one could argue that a ramp-wise incremental rowing GXT is superior for determining VO_2_peak. However, we opted for the typical stepwise test recommended by the German Rowing Association, and to which the athletes were used.

## 5. Conclusions

The inclusion of HIIT in the training program of well-trained young elite rowers increases endurance performance and aerobic capacity. According to the results, HIIT offers a time-efficient and suitable training tool for improving aerobic performance in young elite rowers.

## Figures and Tables

**Table 1:** Participants characteristics at baseline.

Items	Total (*n* = 17)	IG (*n* = 10)	CG (*n* = 7)
Age (years)	15.3 ± 1.26	15.4 ± 1.3	15.3 ± 1.2
Height (cm)	182.9 ± 9.1	183.4 ± 7.7	182.3 ± 11.4
Body mass (kg)	74.5 ± 13.3	74.4 ± 11.7	74.7 ± 16.3
Body mass index (kg/m^2^)	22.1 ± 2.6	22.00 ± 2.9	22.3 ± 2.4
VO_2_peak (mL/kg/min)	59.0 ± 3.9	59.4 ± 2.3	58.4 ± 5.7
Number of light weight rowers (<65 kg)	5	3	2

Values are means ± SD unless stated otherwise.

## Data Availability

The data presented in this study are available on request from the corresponding author.
